# Statistical Trends in the *Journal of the American Medical Association* and Implications for Training across the Continuum of Medical Education

**DOI:** 10.1371/journal.pone.0077301

**Published:** 2013-10-30

**Authors:** Lauren D. Arnold, Melissa Braganza, Rondek Salih, Graham A. Colditz

**Affiliations:** 1 Division of Public Health Sciences, Department of Surgery, Washington University in St. Louis, School of Medicine, St. Louis, Missouri, United States of America; 2 George Warren Brown School of Social Work and the Division of Public Health Sciences, Department of Surgery, School of Medicine, both at Washington University in St. Louis, St. Louis, Missouri, United States of America; 3 Division of Public Health Sciences, Department of Surgery, Washington University in St. Louis, School of Medicine, St. Louis, Missouri, United States of America; National Taiwan University, Taiwan

## Abstract

**Background:**

Statistical training across the continuum of medical education may not have advanced at the pace of statistical reporting in the medical literature, yet a comprehensive understanding of statistical concepts most commonly presented in current research is critical to the effective practice of Evidence Based Medicine. The objective of this content analysis was to describe statistical techniques used in a leading medical journal, *JAMA*, across a 20-year period, with a focus on implications for medical education.

**Methods and Findings:**

Two issues of *JAMA* published each month in 1990, 2000, and 2010 were randomly selected; from these, 361 articles were reviewed. Primary focus, study design, and statistical components were abstracted and examined by year of publication. The number of published RCTs and cohort studies differed significantly across years of interest, with an increasing trend of publication. The most commonly reported statistics over the 20-year period of interest included measures of morbidity and mortality, descriptive statistics, and epidemiologic outcomes. However, between 1990 and 2010, there was an increase in reporting of more advanced methods, such as multivariable regression, multilevel modeling, survival analysis, and sensitivity analysis. While this study is limited by a focus on one specific journal, a strength is that the journal examined is widely read by a range of clinical specialties and is considered a leading journal in the medical field, setting standards for published research.

**Conclusions:**

The increases in frequency and complexity of statistical reporting in the literature over the past two decades may suggest that moving beyond basic statistical concepts to a more comprehensive understanding of statistical methods is an important component of clinicians' ability to effectively read and use the medical research. These findings provide information to consider as medical schools and graduate medical education training programs review and revise their statistical training components.

## Introduction

Teaching and using statistics across the spectrum of medical training is a key issue in medical education today. Much of the recent attention relates to the impending addition of statistics questions to the Medical College Admissions Test (MCAT) 2015, required for admission by most U.S. medical schools, signaling a shift in focus in medical school preparation from the traditional premedical sciences to other aspects of population health [1]. These changes parallel earlier calls by the Institute of Medicine [2] and the Association of American Medical Colleges (AAMC) [3] to integrate principles of population health – including statistics – across the continuum of medical education. Underscoring this need is the emphasis that medical education places on evidence based medicine (EBM), teaching medical students, residents, and fellows to critically evaluate the literature and use this evidence in conjunction with clinical expertise to make diagnostic and management/treatment decisions [4]. Integral to the appropriate and effective use of the literature is physician numeracy [5], or moving beyond familiarity with and recognition of statistical terms to achieving a solid understanding of the statistical components of research studies.

While increasing attention has been given to teaching and using statistics in medical education across the continuum of lifelong learning [5], from pre-medical and undergraduate medical education through continuing medical education, it is unclear how well this is being incorporated into training and whether the most relevant and useful concepts are being taught. An examination of statistical components in *New England Journal of Medicine* found that approximately half of articles published in 1978–1979 were accessible with knowledge of only basic descriptive statistics (e.g. percentages, means) [6], [7]; knowledge of t-tests and Chi-Square was estimated to increase access to nearly 75% of articles [7]. While medical education and statistical reporting in the literature have evolved since the late 1970s, they may not have advanced at the same pace. A recent cross-sectional study found that less than half of 277 internal medicine residents surveyed had correct knowledge and interpretation of statistics in the medical literature, with notable deficits in advanced statistics such as Kaplan Meier and regression analysis [8]. This suggests that the level of statistical education in medical training may not be enough to adequately comprehend the broad range of statistics reported in the clinical literature today.

Traditionally, statistics courses have not been part of the required pre-medical curriculum, which focuses largely on the basic biological and physical sciences. Even through the mid-1990s, not every medical school included statistics as part of its medical student curriculum. In 1993, a survey of 100 medical schools found that only 83% offered a statistics course as part of the undergraduate medical curriculum, and none of the schools surveyed required statistics for admission [9]. Nearly two decades later, the 2011–2012 Medical School Admissions Requirements (MSAR) reports that 57 medical schools have a math requirement for admission; only nine of these have a specific statistics prerequisite. Harvard Medical School plans to include statistics as a pre-medical requirement beginning in 2015 [10], [11], and it can be anticipated that others will follow suit to reflect the MCAT 2015 changes. This reflects a shift in emphasis on the quantitative background entering medical students should have and be able to build upon as they embark on their training.

With the renewed interest in statistics as part of medical training comes the question of *what* should be taught and reinforced throughout medical training. Rather than asking *if* future physicians should be required to learn and use statistics, the question becomes “What type and depth of statistics do future physicians *need* to know?” A critical element needed to address these issues is evidence from the medical literature regarding common statistical measures and approaches published today and how this relates to that published in previous decades. Thus, and as a starting point, the current study was a content analysis that reviewed the statistical measures and techniques reported in the *Journal of the American Medical Association (JAMA)* and examined how the nature and use of statistics in the literature has changed over the last 20 years. *JAMA* was specifically chosen due to its reputation for being read by a diverse clinical audience in a range of specialties and for publishing high-quality research that contributes to EBM.

## Methods

**Figure 1:**
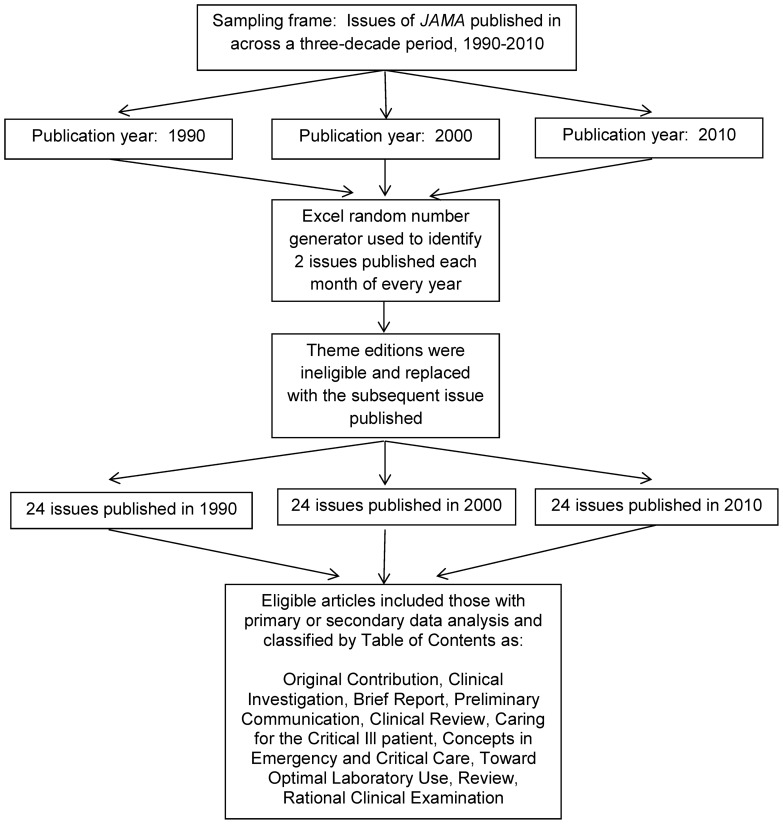
Flow chart outlining selection criteria of articles for content analysis.

To conduct this content analysis, a stratified random sample of Journal issues was identified, and articles published within these issues examined for statistical content (Figure 1). The sampling frame for articles included all issues of the *Journal of the American Medical Association (JAMA)* published in the years 1990, 2000, and 2010. A random number generator in Excel was used to select two weekly issues of JAMA from each month within each year of interest. In situations where a special theme edition was among the weekly issues randomly selected, that issue was excluded (to avoid potential bias in content and statistical analyses presented), and the subsequent issue was selected for review. All articles within those issues selected were then evaluated for eligibility; eligible articles were those in which authors implemented a study and analyzed primary or secondary data. Specifically, the following categories of articles were eligible for inclusion: original contribution, clinical investigation, brief report, preliminary communication, clinical review, caring for the critical ill patient, concepts in emergency and critical care, toward optimal laboratory use, review, and rational clinical examination. As commentary, editorial, clinical crossroads, clinical crossroads update, special communication, and consensus statement articles did not involve primary or secondary data analysis, they were excluded from the study.

A reading schedule was created using the Microsoft Excel random number generator to determine month order for reading. Year order within each month was also randomly determined. For example, January articles might have been read in the order of 1990/2010/2000, while March could be read in the order of 2010/2000/1990. Two readers with masters-level training in public health were assigned data abstraction schedules, in which months were read in the same order, but the year and issue orders within each month were rotated for the purposes of minimizing fatigue bias.

The readers independently abstracted data pertaining to the type of article (as determined by Table of Contents), primary focus, study location, design, data source (s), collection or analysis of biospecimens, laboratory value measurements, power, statistical software, and statistical content. When data did not clearly fall into one of the pre-determined categories (e.g. study design was not clearly specified), coding was discussed and consensus reached.

If no sources of data were mentioned in the article but demographic or background information was collected or presented, data abstracters operated under the assumption that the source of data was self-reported. Power was recorded when an article's authors reported the power for their study; if power was calculated based on the study's anticipated – but not actual – sample size, and thus did not reflect the actual power of the study, then it was not recorded on the data abstraction form. If statistics other than descriptive measures (i.e. multiple regression, Wilcox rank tests, multi-level modeling, or Kaplan Meier curve) were calculated, large sample sizes (e.g. >1,000) were analyzed, or computer-generated graphs were presented in the article but no software package was mentioned, it was recorded as software not otherwise specified. When computer-generated graphs of large sample sizes (e.g. >1,000) were presented in the article but no software package was mentioned, it was assumed that the authors used Excel. If the authors calculated hazard ratios but did not specify the type of survival analysis, the hazard ratios were coded as Cox proportional survival analysis.

The readers entered data into independent files and then merged entries into one file for data reconciliation. Instances of discordant information were flagged, and the readers reconciled the data case-by-case, referencing the article when discrepancies arose. The majority of discrepancies were due to errors in data entry or failure of one reader to abstract the information from the article. When discrepancies could not be solved by referencing the article, the readers discussed the issue and reached agreement; a third investigator was consulted if necessary.

Descriptive statistics were generated for each data category (e.g. statistical test or method), overall and by year of publication. Significant differences for variables over the three study years (1990, 2000, 2010) were examined using Chi-Square, with p<0.05. IBM SPSS Statistics v.19 (Chicago, IL) was used for all analyses.

## Results

A total of 361 articles were reviewed: 133 (36.8%) from 1990, 122 (33.8%) from 2000, and 106 (29.4%) from 2010. The majority of articles were categorized by the Journal as “Original Contribution” (n = 273; 75.6%). As demonstrated in Table 1, the most common study designs published included randomized controlled trials (RCTs) (16.9%, n = 67), cohort studies (15.5%, n = 56), descriptive studies (15.2%, n = 55), and cross-sectional studies (14.4%, n = 52); descriptive studies include categories such as case reports and those in which trends over time were described or compared. There were significant differences in study designs utilized over the three years (1990, 2000, 2010) of publication. The number of published RCTs and cohort studies increased in number over time (1990 and 2010), while cross sectional decreased during the same period. Although less common that other study designs, publication of meta-analyses also increased over time (e.g. 2.4% of studies reviewed from 1990 vs. 16% of studies in 2010).

**Table 1:** Characteristics of 361 articles published in *JAMA*, 1990, 2000, 2010.

		Article Year			
Characteristics	Total	1990 (n = 133)	2000 (n = 122)	2010 (n = 106)	p-value*
Study Design					<0.001
Descriptive^†^	55 (15.2%)	34 (25.6%)	9 (7.4%)	12 (11.3%)	
Cross-sectional	52 (14.4%)	24 (18.0%)	21 (17.2%)	7 (6.6%)	
Case control^‡^	19 (5.3%)	10 (7.5%)	5 (4.1%)	4 (3.8%)	
Cohort	56 (15.5%)	9 (6.8%)	23 (18.9%)	24 (22.6%)	
Meta-analysis/systematic review	25 (6.9%)	3 (2.3%)	5 (4.1%)	17 (16.0%)	
RCT	67 (18.6%)	14 (10.5%)	30 (24.6%)	23 (21.7%)	
Other	87 (24.1)	39 (29.3)	29 (23.8)	19 (17.9)	
Statistical software					
SAS	90 (24.9%)	7 (5.3%)	31 (25.4%)	52 (49.1%)	<0.001
SPSS	23 (6.4%)	3 (2.3%)	6 (4.9%)	14 (13.2%)	0.002
STATA	41 (11.4%)	0 (0.0%)	7 (5.7%)	34 (32.1%)	<0.001
Not specified	152 (42.1%)	90 (83.3%)	61 (52.6%)	1 (1.0%)	<0.001
Biospecimen data	124 (34.3%)	50 (37.6)	44 (36.1)	30 (28.3)	0.287
Lab values used/ measured	130 (36.0%)	54 (40.6)	45 (36.9)	31 (29.2)	0.186

* Chi-square for difference by year; ^†^Includes case studies, comparative studies; ^‡^Inlcudes nested case control.

Reflecting the frequencies of RCT publication, the majority of studies reviewed focused on new therapeutic uses (15.5%, n = 56) (Table 2). This was followed by a focus in “healthcare issues” (12.7%, n = 46), which included studies on cost effectiveness in healthcare, quality of care, and Medicare. Chronic disease studies were the third most common topical focus (11.4%, n = 41); it is noted that heart disease was categorized separately from “chronic disease” due to a large number of studies in this area relative to other chronic illnesses.

**Table 2:** Primary focus of 361 articles published in *JAMA* 1990, 2000, 2010.

Article Focus	Frequency
New therapeutic uses	56 (15.5%)
Healthcare issues*	46 (12.7%)
Chronic disease^†^	41 (11.4%)
Heart disease	34 (9.4%)
Maternal child health^‡^	31 (8.6%)

* Includes topics such as cost-effectiveness of care, quality of care, and Medicare; ^†^Excluding heart disease; includes ALS, asthma, food allergies, sickle cell, and thyroid disorders; ^‡^Includes reproductive/sexual health.

Overall, the most commonly reported statistics in the Journal among the articles reviewed included measures of morbidity and mortality (e.g. incidence, prevalence, mortality), descriptive statistics (e.g. means and percentages), and “low-level” epidemiologic statistics (e.g. standard deviations, standard errors) (Table 3). Power was reported infrequently overall, with significantly differences over time; more studies reported power in 2010 (26.5%, n = 28; p<0.001) as compared to 2000 and 1990. Between 1990 and 2010, there was a significant increase in the reporting of more advanced statistics, specifically sensitivity analysis (49.1% in 2010 vs. 22.6% in 1990), multiple regression (48.1% in 2010 vs. 23.1% in 1990), survival analysis (43.4% in 2010 vs. 14.3% in 1990), multi-level modeling (32.1% in 2010 vs. 2.3% in 1990), intention-to-treat analysis (22.6% of studies in 2010 vs. 4.5% in 1990), and p-trend (13.2% in 2010 vs. 4.5% in 1990).

**Table 3:** Statistical measures and methods in *JAMA* articles published in 1990, 2000, and 2010*.

	Article Year			
Characteristics	1990 (n = 133)	2000 (n = 122)	2010 (n = 106)	p-value
Descriptive statistics	124 (93.2%)	122 (100%)	106 (100%)	-
Low-level statistical measures^†^	108 (81.2%)	116 (95.1%)	105 (99.1%)	<0.001
Morbidity & mortality	76 (57.1%)	60 (49.2%)	73 (68.9%)	0.011
ANOVA	26 (19.5%)	24 (19.7%)	18 (17.0)	0.844
Chi square	54 (40.6%)	51 (41.8%)	51 (48.1%)	0.471
Fisher exact	19 (14.3%)	18 (14.8%)	20 (18.9%)	0.583
Mantel-Haenszel	11 (8.3%)	15 (12.3%)	7 (6.6%)	0.301
Epidemiologic statistics^‡^	28 (21.1%)	34 (27.9%)	33 (31.1%)	0.190
t-test	28 (21.1%)	31 (25.4%)	28 (26.4%)	0.577
Power	7 (5.3%)	7 (5.7%)	28 (26.4%)	<0.001
p-trend	6 (4.5%)	17 (13.9%)	14 (13.2%)	0.023
Pearson correlation coefficient	13 (9.8%)	10 (8.2%)	5 (4.7%)	0.340
Logistic regression	27 (20.3%)	42 (34.4%)	28 (26.4%)	0.039
Simple linear regression	12 (9.0%)	17 (13.9%)	13 (12.3%)	0.460
Poisson regression	0 (0.0%)	11 (9.0%)	8 (7.5%)	0.003
Log-rank test	2 (1.5%)	9 (7.4%)	15 (14.2%)	0.001
Multi-level modeling	3 (2.3%)	11 (9.0%)	34 (32.1%)	<0.001
Multiple comparison	7 (5.3%)	8 (6.6%)	9 (8.5%)	0.609
Multiple regression	32 (24.1%)	52 (42.6%)	51 (48.1%)	<0.001
Non parametric test	17 (12.8%)	19 (15.6%)	23 (21.7%)	0.173
Wilcoxon Rank	13 (9.8%)	14 (11.5%)	19 (17.9%)	0.150
Survival analysis	19 (14.3%)	27 (22.1%)	46 (43.4%)	<0.001
Cox models	10 (7.5%)	17 (13.9%)	34 (32.1%)	<0.001
Kaplan Meier	5 (3.8%)	13 (10.7%)	24 (22.6%)	<0.001
Sensitivity analysis	30 (22.6%)	44 (36.1%)	52 (49.1%)	<0.001
Intention to treat	6 (4.5%)	18 (14.8%)	24 (22.6%)	<0.001
Transformation	9 (6.8%)	12 (9.8%)	10 (9.4%)	0.6374

* Excludes statistics in which there were n<15 across all three years of review; Includes standard deviations, standard errors, confidence intervals, and p-values; ^‡^Includes odds ratios, relative risks, attributable risk, sensitivity, and specificity.

## Discussion

Because medical education and standards for publication are continuously evolving, it is necessary to revisit the issue of statistical reporting to see if findings from the 1980s and 1990s are still supported today. In this way, our study adds to the literature as it supports the continuing use of more advanced statistical measures and techniques in the medical literature. In turn, this suggests higher levels of statistical understanding may be needed in order for clinicians to effectively use some – but not necessarily all – of the literature in the practice of Evidence Based Medicine today, relative to a decade or two ago. In this way, this study provides additional evidence to support re-visiting of what is taught to medical students, residents, and fellows throughout their training to, familiarize them with emerging statistical methods.

More specifically, we observed that nearly all studies published in 1990, 2000, and 2010 included some form of statistical reporting and that more complex multivariable regression methods (e.g., linear, Cox proportional hazards), and contingency tables were present in up to 50% of papers published in *JAMA* in 2010. Furthermore the proportion of papers reporting multilevel modeling results, multivariable regression, survival analysis, and sensitivity analysis, all increased with significant differences over the study period of 1990 to 2010. These higher level statistical methods require a solid statistical understanding to interpret their application and the results reported. Traditional epidemiologic study design (case-control and cohort), meta-analysis, and randomized controlled trials account for 60% of study designs by 2010, with a corresponding decline in the proportion of studies that were descriptive or cross sectional. It is possible that some of these trends and techniques, such as intention-to-treat analysis were performed in earlier studies but were less likely to be included in studies published before implementation of CONSORT guidelines in 1996. Nonetheless, the trends observed imply that understanding the strengths and limitations and applicability of results from varied research designs and research synthesis is required to interpret and apply results of studies reported in *JAMA* as of 2010.

These findings provide information to consider as medical schools and post-graduate training programs review and revise statistical training components. Moreover, for clinicians and medical investigators interested in continuing their own education in statistics, the frequencies in Table 3 may provide a guide for the techniques that learners and faculty alike should consider mastering in order to comprehensively and critically use high-quality literature as part of the practice of Evidence Based Medicine. As the structure of the 2015 MCAT encourages students to gain foundational knowledge in statistics during their premedical education, and as proposed changes to add a statistics course to the premedical requirements are debated [12], undergraduate medical education will need to consider revisions to their pre-clinical and clinical curricula. In turn, this will affect graduate and postgraduate medical education. This downstream effect that begins with a pre-medical focus will in turn allow medical schools and post-graduate training programs to expand on foundational statistical knowledge and provide a more comprehensive approach to statistical and epidemiologic training [12].

The focus on one journal for this content analysis is a limitation as it restricts generalizability of findings and does not account for variation by specialty. For example, preferred choice of study design (s) and data analysis expectations in surgical fields may differ from those in psychiatry or pediatrics. Thus, trends in study design and analytic techniques presented here may differ from journals with more directed target audiences and areas of focus. If this content analysis of *JAMA* articles was extended to include articles from other comparable journals (e.g. similar impact factors, area of focus, target audience), such as *New England Journal of Medicine*, or if additional articles from *JAMA* were included in the sample, then it is anticipated that individual findings would vary but that overall trends of increasing statistical complexity over the decades would be similar.

One journal alone does not serve as a “gold standard” by which to judge medical education needs. However, the choice of Journal for this study does serves as a strength in that the study focused on a leading medical journal with extremely broad readership. The Journal is widely read by clinicians in a variety of specialties and publishes across a range of clinically related issues. In these ways, understanding the statistical measures and techniques most commonly reported in JAMA may serve as a once source of evidence for generating discussion about and guiding areas on which to focus the statistical aspects of medical education.

Although others have reported on use of statistics in specific disciplines, many of these studies are older in nature or have used smaller samples of articles. Results from the *New England Journal of Medicine* suggest that by 2004 nearly 88% of articles required some statistical analysis beyond descriptive statistics [13]. To assess differences in the use of statistical methods in general medicine journals and specialized journals, we identified reviews of statistical methods used in specialized journals. A 1995 study comparing prevalence and use of statistical analysis found that rheumatology journals [14] tended to use fewer and simpler statistics than general medicine journals. A 1994 study of use of statistical analytic methods in the *American Journal of Radiology* and *Radiology* [15] shows that 44% of the major articles used no statistical methods or descriptive statistics only, reflecting the nature of imaging studies; 15% used only two methods, and 14% used three or more methods. Similar results were reported for *Clinical Radiology* and *British Journal of Radiology* [16]. On the other hand, a review of all papers in volume 115 of *Pediatrics* demonstrated that statistical complexity increased from 1982 to 2005. The number of statistical procedures per article increased (to 3.9 in 2005 from 2.5 in 1982), as well as the range of inferential statistical procedures used during that time [17]. A 1987 review of surgical journals [18] illustrated that a reader with knowledge of descriptive statistics had access to 44.5% of the articles, whereas in 2002, only 18% of the articles in obstetrics and gynecology journals [19] did not use any type of statistical method, and by 2005 only 11% of general pediatrics articles did not use inferential statistics. In anesthesiology, similar results are reported from 3 leading journals, which in 2004 required more than descriptive statistics to access 75% of articles [20]. The combination of this evidence points to the use of increasingly complex statistical techniques in both general and special medical journals, again underscoring the need for understanding of such methods in order to adequately read, interpret, and use study results presented in some of the medical literature.

Over the time frame of this study of use of statistics, 1990 to 2010, we note that there has been little change in content of medical education [12], and in the way evidence-based medicine is taught [5]. Even in instances where statistical content of training may have been revised and updated, the degree to which material is covered may be limited [5]. This contrasts with the substantial increases in frequency and complexity of statistical reporting. While a limitation of this focus on frequency of use may ignore more specific details of sample selection and approaches to control for bias – factors that are important when considering evaluation and applicability of evidence for practice, we assume that understanding the underlying design and statistical methods is necessary before moving to applicability of results.

Findings from this content analysis of JAMA articles over a three-decade period support and extend trends in previous studies that point to continued use of complex statistical approaches, such as multivariate modeling and regression, in the medical literature. While this is not generalizable to all the medical literature, *JAMA* is considered a leading Journal in the field, and the studies published are read by a broad range of clinicians. While our findings do not directly suggest that medical education necessarily needs to be modified, the statistical reporting trends described may have implications for medical education. Similarly, while this study does not provide data to suggest that improved statistical knowledge could translate to more effective use of the literature, we do propose that physicians' familiarity with certain (advanced) statistical approaches may assist them in critically evaluating and weighing the literature. The evolution of statistical software programs over the past years has expanded analytic capabilities – or at least broadened the spectrum of appropriate statistical options. To this end, medical educators may wish to be aware of the benefits and limitations of different and more complex statistical strategies as they try to teach certain topical content or critical evaluation skills. Moreover, as future and current clinicians engage in a life-long learning process, findings from this study may be used as part of the discussion about statistical training across the continuum of medical education.

## References

[1] AAMC (2011) MCAT^2015^ A better test for tomorrow's doctors: Preview Guide for MCAT^2015^. Washington, D.C.: Association of American Medical Colleges. 149 p.

[2] Gebbie KM, Rosenstock L, Hernandez LM, Institute of Medicine (U.S.), Committee on Educating Public Health Professionals for the 21st Century (2003) Who will keep the public healthy?: Educating public health professionals for the 21st century. Washington, D.C.: National Academy Press. 304 p. 25057636

[3] Sabharwal R (2002) Trends in medical school graduates' perceptions of instruction in population-based medicine. AAMC: Analysis in Brief 2:1. Available: https://www.aamc.org/download/102332/data/aibvol2no1.pdf. Accessed 2013 September 19.

[4] Sackett DL, Rosenberg WM, Gray JA, Haynes RB, Richardson WS (1996) Evidence based medicine: what it is and what it isn't. BMJ 312: 71–72. 8555924 10.1136/bmj.312.7023.71 PMC2349778

[5] Rao G, Kanter SL (2010) Physician numeracy as the basis for an evidence-based medicine curriculum. Acad Med 85: 1794–1799. 20671540 10.1097/ACM.0b013e3181e7218c

[6] Colditz GA, Emerson JD (1985) The statistical content of published medical research: some implications for biomedical education. Med Educ 19: 248–255. 4010572 10.1111/j.1365-2923.1985.tb01315.x

[7] Emerson JD, Colditz GA (1983) Use of statistical analysis in the New England Journal of Medicine. N Engl J Med 309: 709–713. 6888443 10.1056/NEJM198309223091206

[8] Windish DM, Huot SJ, Green ML (2007) Medicine residents' understanding of the biostatistics and results in the medical literature. JAMA 298: 1010–1022. 17785646 10.1001/jama.298.9.1010

[9] Looney SW, Grady CS, Steiner RP (1998) An update on biostatistics requirements in U.S. medical schools. Acad Med 73: 92–94. 9447208 10.1097/00001888-199801000-00018

[10] Bryn Mawr Health Professions Advising Office (2012) Medical Schools with Math Requirements. Swathmore University. Available: http://www.swarthmore.edu/Documents/slife/pre_med/Math_Req_for_Medical_School.pdf. Accessed 2012 May 14.

[11] AAMC (2010) Medical school admissions requirements 2011–2012. Washington, D.C.: Association of American Medical Colleges Publication Department. 438 p.

[12] Lambert DR, Lurie SJ, Lyness JM, Ward DS (2010) Standardizing and personalizing science in medical education. Acad Med 85: 356–362. 20107368 10.1097/ACM.0b013e3181c87f73

[13] Agrawal S, Colditz G, Emerson J (2009) Use of statistical analyiss in the New England Journal of Medicine. In: Bailar III J, Hoaglin D, editors. Medical Uses of Statistics. 3rd Edition ed. Hoboken, NJ: John Wiley & Sons. 41–49.

[14] Cardiel MH, Goldsmith CH (1995) Type of statistical techniques in rheumatology and internal medicine journals. Rev Invest Clin 47(3): 197–201. 7569363

[15] Elster AD (1994) Use of statistical analysis in the AJR and Radiology: frequency, methods, and subspecialty differences. AJR Am J Roentgenol 163: 711–715. 8079874 10.2214/ajr.163.3.8079874

[16] Goldin J, Zhu W, Sayre JW (1996) A review of the statistical analysis used in papers published in Clinical Radiology and British Journal of Radiology. Clinical radiology 51: 47–50. 8549048 10.1016/s0009-9260(96)80219-4

[17] Hellems MA, Gurka MJ, Hayden GF (2007) Statistical literacy for readers of Pediatrics: a moving target. Pediatrics 119: 1083–1088. 17545374 10.1542/peds.2006-2330

[18] Reznick RK, Dawson-Saunders E, Folse JR (1987) A rationale for the teaching of statistics to surgical residents. Surgery 101: 611–617. 3576452

[19] Welch GE 2nd, Gabbe SG (2002) Statistics usage in the American Journal of Obstetrics and Gynecology: has anything changed? Am J Obstet Gynecol 186: 584–586; discussion 339. 10.1067/mob.2002.122144 11904628

[20] Rubio Garcia B, Rodriguez Zazo A, Martinez Terrer T, Rubio Calvo E (2010) Use of statistics and the accessibility of original articles published in 3 anesthesiology journals. Rev Esp Anestesiol Reanim 57: 281–287. 20527342 10.1016/s0034-9356(10)70228-0

